# Clinical Variability of GLUT1DS

**DOI:** 10.15844/pedneurbriefs-29-2-5

**Published:** 2015-02

**Authors:** Anastasia Martinez-Esteve Melnikova, Christian M. Korff

**Affiliations:** 1 Pediatric Neurology, Child and Adolescent Department, University Hospitals, Geneva, Switzerland

**Keywords:** Epilepsy, GLUT1 deficiency syndrome, Ketogenic diet, Paroxysmal exercise-induced dyskinesia, SLC2A1 gene

## Abstract

Investigators from Pavia, Rho, Brescia and Milan, Italy, studied 22 patients diagnosed with GLUT1 deficiency syndrome (GLUT1DS) to document clinical or genetic differences between patients with familial *SLC2A1* gene mutations (n=11) and those with sporadic mutations (n=11).

Investigators from Pavia, Rho, Brescia and Milan, Italy, studied 22 patients diagnosed with GLUT1 deficiency syndrome (GLUT1DS) to document clinical or genetic differences between patients with familial *SLC2A1* gene mutations (n=11) and those with sporadic mutations (n=11). Direct gene sequencing in sporadic cases revealed 7 missense, 3 nonsense, and 1 splice site mutation. In the group with familial inheritance, all patients presented with missense mutations. Important differences were observed regarding clinical features. Overall, patients with sporadic mutations had a more severe phenotype than those with familial inheritance. They had more severe intellectual disability, earlier epilepsy onset and greater tendency to be refractory to AEDs treatment and more disabling movement disorders. In the familial group, relatives carrying *SLC2A1* mutations presented with heterogeneous clinical features of variable severity. Furthermore, two patients with genetically confirmed GLUT1DS had siblings with a similar type of epilepsy but without *SLC2A1* gene mutations.

The milder phenotype observed in the familial group, and the reported phenotypic variability among family relatives, confirms the heterogeneity of the clinical expression of *SLC2A1* mutations. This raises the question of the incidence of GLUT1DS, using a ketogenic diet in less symptomatic patients, and genetic counseling concerns. Authors also suggest that symptomatic patients negative for *SLC2A1* mutations should undergo screening in order to discover potential additional pathogenic genes. [1]

COMMENTARY. Since De Vivo first described the classic phenotype in 1991 in two sporadic cases with early-onset epilepsy, developmental delay and acquired microcephaly, the clinical presentation of GLUT1DS has widely expanded. The non-classic phenotypes include patients with isolated movement disorders and mild development delay, those with paroxysmal exertion-induced dyskinesia with epilepsy and those with a so-called carbohydrate-responsive phenotype [2]. Refractory and early-onset absence seizures have also been associated with GLUT1DS [3]. The study of familial cases supports an autosomal dominant inheritance [4], although recessive inheritance has also been reported.

The phenotypic heterogeneity may be partially explained by the underlying genotype. For instance, Leen et al. observed that missense mutations were more frequently responsible for a “mild” phenotype [5, 6]. The non-genetic mechanisms potentially involved include defects in RNA transcription, translation or protein generation. Dysfunction of different mechanisms regulating glucose transport across tissue barriers may also be present [7].

Patients with a high clinical suspicion and negative SLC2A1 mutation, or those with different clinical expression within the same affected family, are particularly challenging. Other genes associated with GLUT1DS, still to be discovered, could contribute to GLUT1DS.

GLUT1DS being a treatable disorder, all efforts should be made to study its clinical variability and facilitate its early diagnosis. This is particularly true for patients with a non-classic phenotype, who are likely to be currently under-diagnosed. Further investigations on other genetic or non-genetic underlying pathophysiological mechanisms should be encouraged.
